# WBC Count vs. CRP Level in Laboratory Markers and USG vs. CT Abdomen in Imaging Modalities: A Retrospective Study in the United Arab Emirates to Determine Which Are the Better Diagnostic Tools for Acute Appendicitis

**DOI:** 10.7759/cureus.47454

**Published:** 2023-10-22

**Authors:** Maryam Risla Shahul Hameed, Siddiqua Shahul Hameed, Reshme Rafi Ahamed, Faiba A Jacob, Biji George

**Affiliations:** 1 General Surgery, Ashford and St. Peters NHS Foundation Trust, Surrey, GBR; 2 Geriatrics/Palliative Care, Magenta Home Health, Dubai, ARE; 3 Obstetrics and Gynecology, Corniche Hospital, Abu Dhabi, ARE; 4 Psychiatry, RAK (Ras Al Khaimah) College of Medicine, Ras Al Khaimah, ARE; 5 Surgery, RAK (Ras Al Khaimah) College of Medicine, Ras Al Khaimah, ARE

**Keywords:** diagnosis of acute appendicitis, laboratory markers, c-reactive protein (crp), acute surgical abdomen, crp in assessment of acute abdomen, wcc in assessment of acute abdomen, abdominal ultrasonography, computed tomography (ct ), white blood cells, acute appendicitis diagnosis

## Abstract

Introduction

Acute Appendicitis (AA) is the most common surgical emergency. Despite the use of various diagnostic parameters, the rate of negative appendectomy remains high (30%). Thus, through our retrospective study, we aim to analyse whether white blood cell (WBC) count or C-reactive protein (CRP) level is more indicative in the confirmation of AA. We also analysed imaging modalities ultrasonography (USG) and computed tomography (CT) of the abdomen to find out which is better for diagnosing AA.

Methods

Patients with suspected AA admitted for laparoscopic appendectomy in Saqr Hospital, Ras Al Khaimah, United Arab Emirates, during 2019-2020 were included in the study. Patients who had either or both WBC and CRP values were included in the study and their diagnosis was confirmed based on histological appendectomy findings. Data analysis was done using IBM SPSS Statistics for Windows, Version 23.0 (Released 2015; IBM Corp., Armonk, New York, United States), receiver operating characteristic (ROC) curve, and chi-square test as required. p-value of <0.05 was considered statistically significant.

Results

Out of the 320 patients with suspected AA, WBC had a p-value of 0.8 (insignificant). A total of 228 patients who had elevated WBC had confirmed histological diagnosis of AA, and 152 patients who were tested for CRP and had elevated levels had confirmed histological diagnosis of AA. CRP had a p-value of 0.04 (significant). However, when the ROC curve was used as evidence to see which was a better test, WBC and CRP both had a low area under the curve (AUC), which proved that they were not the most accurate diagnostic marker in diagnosing AA. However, CRP was slightly better than WBC.

A total of 266 patients underwent USG abdomen and it had a p-value of 0.4 (insignificant), while 118 patients underwent CT scan, which had a p-value of 0.01 (significant). CT abdomen was statistically proven as a better radiological investigation. Also, when the ROC curve was used to compare USG and CT abdomen, CT again proved to be a better radio diagnostic method for AA.

Conclusions

From our study, it can be concluded that CRP is better than WBC in ruling in appendicitis and CT abdomen is better than USG in diagnosing appendicitis, but CT abdomen is only next to histological diagnosis in confirming AA. Hence, we recommend doing CRP as the primary laboratory marker for suspected cases of AA. CT abdomen is the ideal imaging modality in cases of suspected AA where clinical examination, laboratory values, and ultrasound examination are inconclusive.

## Introduction

Acute appendicitis (AA) is the most common surgical condition that presents with an acute abdomen needing emergency surgery [1]. The lifetime risk of AA in the general population is about 7% [2]. Correctly diagnosing AA remains a challenge [1]. The typical clinical picture is unfortunately found in much fewer patients than it is thought and too much reliance on laboratory findings can misguide a surgeon’s diagnosis [2]. Despite all diagnostic methods including history, physical examination, laboratory investigations, clinical scores like Alvarado scores, and radiological methods like ultrasonography (USG) and computed tomography (CT), negative appendectomy rates still have been reported up to 30% in the literature, with an average percentage of 15-30% [3,4]. In practice, the diagnosis of AA is supported by the presence of elevated inflammatory markers, namely, white blood cell count (WCC) and C-reactive protein (CRP). However, some studies have shown that neither of these markers is diagnostic nor specific for AA [5]. We conducted this retrospective study with the intention of analysing which one of the two laboratory investigations, i.e., WCC and CRP, is more indicative in confirming the diagnosis. We have also analysed the imaging modalities of USG and CT abdomen to find which is a better tool for diagnosing AA.

## Materials and methods

In this retrospective study, we included 320 patients both male and female admitted to Saqr Hospital, Ras Al Khaimah (RAK), United Arab Emirates, between September 2019 and September 2020, suspected to have AA and who had either or both WBC and CRP values available. We excluded patients who were immunocompromised, on steroids, with active infection other than suspected appendicitis, or with an operative diagnosis other than AA. The study was approved by the United Arab Emirates Ministry of Health and Prevention, Research Ethics Committee/RAK Subcommittee on 7/5/2020 (approval number: MOHAP/REC/2020/32-2020-UG-M).

Laboratory tests were performed on patients on admission to the hospital before starting medications. For reference, the normal values for WBC and CRP were taken as 4-11x10^9^/L and less than 10 mg/L, respectively.

For statistical analysis, we used IBM SPSS Statistics for Windows, Version 23.0 (Released 2015; IBM Corp., Armonk, New York, United States). We grouped the categorical variables as strings for patients' personal data and as numeric for the WBC and CRP values. To compare the diagnostic accuracy between WBC count and CRP levels, we used the area under the receiver operating characteristic (ROC) curve (AUC). A similar method was used for comparing imaging modalities USG and CT abdomen. We derived the sensitivity, specificity, positive predictive value, and negative predictive value of all the variables using cross-tabulations. A chi-square test was used to analyse the association between the two variables. p-value of <0.05 was considered statistically significant.

## Results

The study population consisted of 320 patients who presented with symptoms that were characteristic of AA. Most of the population was male comprising 211 individuals and the remaining 109 were females. There was no specific age limit taken into consideration and the range of age we had in our study population was 6-90 years. The study population was divided into four groups as follows: Group 1 consisted of patients who had elevated WBC, Group 2 consisted of patients who had elevated CRP, Group 3 consisted of patients who had undergone CT Abdomen, and Group 4 consisted of patients who had undergone USG Abdomen. After appendectomy, it was observed which was a better diagnostic marker for AA.

In patients with AA, WBC for 320 patients were tested. The diagnostic parameters for WBC counts were as follows: when the cut-off value taken into consideration was >10 × 10^9^/L, the highest degree of sensitivity was 75%, specificity was 77%, PPV was 92%, and NPV was 91%. The percentage data on WBC are mentioned in Table 1.

**Table 1:** Elevated WBC compared with histological diagnosis of acute appendicitis

			Histological Diagnosis		Total
			Normal	Appendicitis	
WBC COUNT	Normal WBC	N (%)	6 (1.87%)	68 (21.25%)	74 (23.12%)
	Elevated WBC	N (%)	18 (5.62%)	228 (71.25%)	246 (76.87%)
Total		N (%)	24 (7.5%)	296 (92.5%)	320 (100%)

A total of 152 patients out of 263 who were tested for CRP and had elevated levels had confirmed histological diagnosis of appendicitis. The diagnostic parameters for CRP values were as follows: when cut-off value of ≥3 mg/L was taken into consideration, the highest degree of sensitivity was 85%, specificity was 62%, PPV was 89%, NPV was 96%. The percentage data on CRP are mentioned in Table 2.

**Table 2:** Elevated CRP compared with histological diagnosis of acute appendicitis CRP: C-reactive protein

			Histological Diagnosis		Total
			Normal	Appendicitis	
CRP Levels	Normal	N (%)	3 (1.14%)	91 (34.6%)	94 (35.74%)
	Elevated	N (%)	17 (6.46%)	152 (57.79%)	169 (64.25%)
Total		N (%)	20 (7.6%)	243 (92.39)	263 (100%)

**Figure 1:**
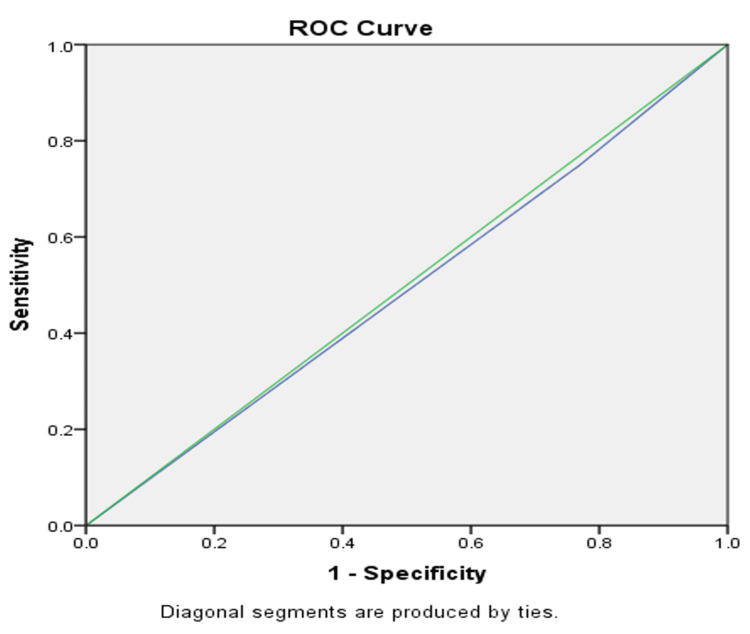
ROC curve for elevated WBC. The green diagonal line is the reference line and the blue diagonal line depicts WBC count; the line depicting WBC falls slightly below the reference line. ROC: receiver operating characteristic

**Figure 2:**
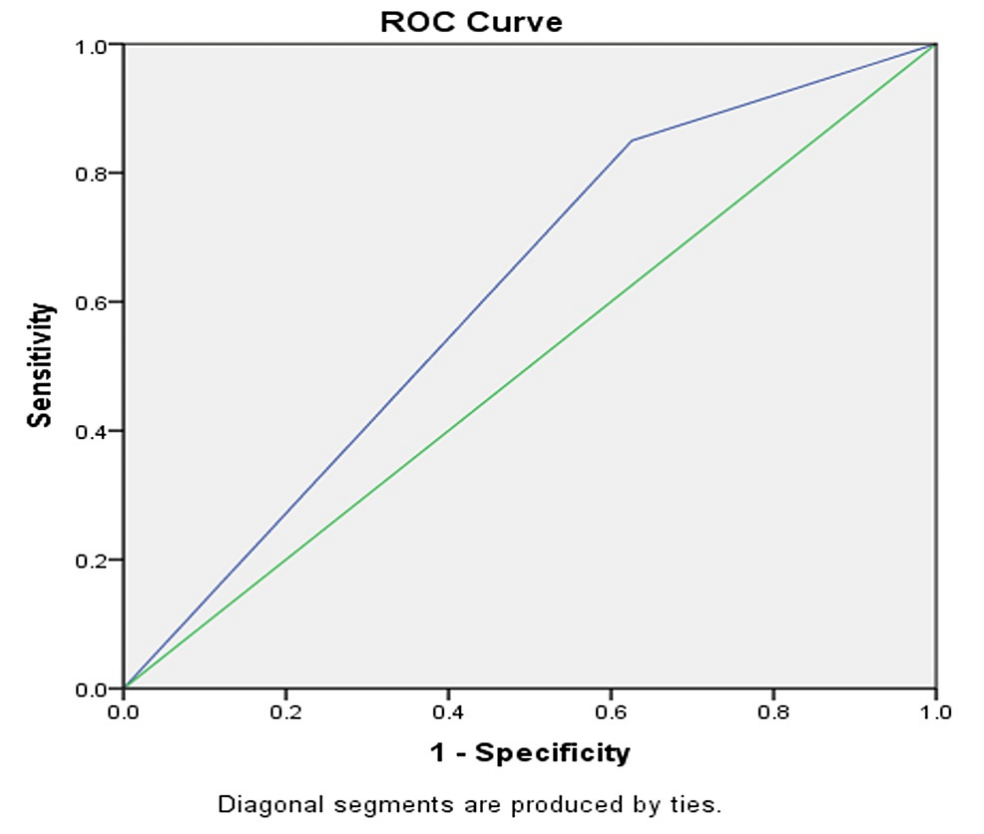
ROC curve for elevated CRP. The green diagonal line is the reference line and the blue diagonal line depicts CRP; the line depicting CRP is above the reference line. ROC: receiver operating characteristic; CRP: C-reactive protein

As depicted in Tables 1-2, we can conclude that the sensitivity of CRP is higher than WBC, which makes it a better test for screening and ruling in appendicitis. Additionally, the NPV of CRP is higher than WBC, which makes it a better test compared to WBC in ruling out appendicitis. However, when the ROC curve was used as evidence to see which one was a better test, WBC and CRP were plotted on the graph and both had low AUC, which proves that neither are the most accurate diagnostic marker. We can, however, see that CRP is slightly better than WBC (Figures 1-2). Mean WBC had a statistically insignificant value p=0.8 and CRP values were statistically significant p=0.04.

A total of 266 patients out of 320 underwent USG abdomen. Diagnostic parameters for abdominal USG were as follows: when the appendix was dilated >6mm in diameter, non-compressible and inflamed with peri-appendiceal fluid collection was taken into consideration. The highest degree of sensitivity was 59%, specificity was 67%, PPV was 92%, and NPV was 89%. The percentage data on USG are mentioned in Table 3.

**Table 3:** USG diagnosis compared with histological diagnosis of acute appendicitis

			Histological Diagnosis		Total
			Normal	Appendicitis	
USG Diagnosis	Negative	N (%)	9 (3.38%)	79 (29.69%)	88 (33.08%)
	Appendicitis	N (%)	13 (4.88%)	165 (62.03%)	178 (66.91%)
Total		N (%)	22 (8.27%)	244 (91.72%)	266 (100%)

A total of 118 patients out of 320 underwent CT abdomen. The diagnostic parameters for CT abdomen were as follows: appendiceal dilatation (>6 mm diameter), wall thickening (>3 mm), and enhancement, presence of appendicolith, peri-appendiceal inflammation (fat stranding, thickening of mesoappendix, extraluminal fluid, inflammatory mass), abscess, and gangrenous appendicitis were all taken into consideration. The highest degree of sensitivity was 38%, specificity was 83%, PPV was 94%, and NPV was 68%. It also had a statistically significant p-value of 0.01. The percentage data on CT scans are mentioned in Table 4. USG had a statistically insignificant value of 0.4 compared to CT, which had a p-value of 0.01. Thus, CT abdomen is superior to USG abdomen in diagnosing acute appendicitis.

**Table 4:** CT diagnosis compared with histological diagnosis of acute appendicitis

			Histological Diagnosis		Total
			Normal	Appendicitis	
CT Abdomen Diagnosis	Normal	N(%)	8 (6.77%)	17 (14.4%)	25 (21.18%)
	Appendicitis	N(%)	5 (4.23%)	88 (74.57%)	93 (78.81%)
Total		N(%)	13 (11.01%)	105 (88.98%)	118 (100%)

Table 5 compares laboratory and radiological investigations.

**Table 5:** Comparison of laboratory and radiological investigations

Paramneters	WBC	CRP	USG	CT
Sensitivity	75%	85%	59%	38%
Specificity	77%	62%	67%	83%
Positive predictive value	92%	89%	92%	94%
Negative predictive value	91%	96%	89%	68%

Therefore, from the above data acquired, it can be concluded that CRP is better than WBC in ruling in appendicitis. At the same time, CT abdomen is next only to histological diagnosis in confirming AA.

## Discussion

AA is one of the most common causes of acute abdomen. More than 300 patients presented to the hospital with AA requiring laparoscopic appendectomy between September 2019 and September 2020. In most cases, the patients were symptomatic, and they required laboratory and radiological tests to support the diagnosis.

WBC is commonly used for confirming any suspected inflammation or infection in the body. Fatima et al., in 2021, revealed that the accuracy of total leucocyte count > 11,000/mm^3^ (as a diagnostic tool) was found to be 82.94%, with sensitivity and specificity being 83.10% and 82.14%, respectively [6]. A study conducted in India by Shafi et al. in 2009 showed the sensitivity and specificity of WBC as 97.82% and 55.55%, respectively [7]. Similarly, Buyukbese Sarsu et al., in 2016, revealed a sensitivity and specificity of 73.4% and 80.0%, respectively in paediatric appendicitis [8].

CRP monitoring enhances the diagnostic accuracy of AA. In a study conducted in the United Kingdom, CRP provides the highest diagnostic accuracy for perforated appendix more than non-perforated AA. A paper presented by Shafi et al. in 2009 showed the sensitivity and specificity of CRP as 95.6% and 77.77%, respectively [7]. Likewise, Buyukbese Sarsu et al. presented a study that was conducted in Turkey in the paediatric age group revealed a sensitivity and specificity of 70.9% and 68.7%, respectively [8].

USG is initially used to aid in the diagnosis of AA, especially in pregnant women, children, and the elderly. It is also used to rule out gynaecological pathologies in women. However, it is highly user-dependent. In a study conducted by Giljaca et al. involving 3306 references identified through electronic searches, 17 reports met the inclusion criteria, with 2841 included participants [9]. The summary sensitivity and specificity of USG for the diagnosis of AA were 69% and 81%, respectively. The study also concluded that USG does not contribute the maximum in diagnosing AA in suspected patients. Hence, they need to be referred to higher specific diagnostic procedures. In another study by Fu et al., from 3,193 references, a total of 18 studies were selected. Overall sensitivity of 77.2% and specificity of 60% were observed [10].

CT scan is used as an initial diagnostic or confirmatory tool for AA. It is highly accurate in diagnosing AA, especially when other inflammatory markers are normal, and the symptoms of the patients are not suggestive of AA. It can aid in the diagnosis of dangerous cases like perforated appendicitis, which requires immediate surgical intervention. In a study conducted by Mawiah et al. in King Fahad Specialty Hospital in Saudi Arabia, CT abdomen was shown to have a sensitivity of 86% and a specificity of 16.7% for the diagnosis of AA. Almost 200 patients were included in the study out of which 187 patients were diagnosed as AA through histopathology. Of the 187 patients, 57 patients performed CT scans and 54 patients performed USG. CT scans correctly diagnosed 86% of patients with AA and incorrectly diagnosed 14% of patients with a negative diagnosis of AA [11].

In a study by Daniels et al., USG and CT scans were done in 120 patients with AA to compare the accuracy of the diagnosis of AA using each test. The results were correlated with surgical and histopathological findings at appendicectomy or clinical follow-up. Ninety-three patients had acute appendicitis and 27 patients did not. The sensitivity of CT was 95% and that of USG was 87%. The corresponding specificities were 89% and 74%, respectively, thereby proving CT is more sensitive and specific than USG in patients suspected of having AA, but in whom the presentation is equivocal [12].

In our study, the sensitivity of WBC was 75% and specificity 77%. The sensitivity and specificity of CRP were 85% and 62%, respectively. Of the study population of 320 patients, a total of 266 patients underwent USG and 118 patients had CT abdomen in the present study. USG had a sensitivity of 59% and a specificity of 67%, while CT scans had a sensitivity of 38% and a specificity of 83%. Also, USG had a statistically insignificant value of 0.4 compared to CT, which had a p-value of 0.01.

The limitations of our study are that not all four investigations including WBC count, CRP level, USG and CT abdomen were done in all of the 320 patients. It was based on the clinical judgement of the doctor who had seen the patient on admission. Another key limitation is that the study is retrospective and we cannot go back in the past to have things done differently or observe directly. Although the total number of cases included in the study is 320, it could have been more if not for the time constraints. We had a limited time period of a few hours once a week to collect our data. We had access to data from only one computer at the hospital and it was carried out confidentially in the Consultant's office who supervised us during the study. We managed to get a good amount of data by being consistent and taking turns collecting the data each week. There were no major concerns during the study.

## Conclusions

On examining the ROC curves, it can be concluded that CRP is better than WBC in confirming AA. The difference was statistically significant. CT abdomen is superior to USG abdomen in diagnosing AA. The accuracy of CT abdomen in the diagnosis of AA is high; however, operative/ histological features of AA might be seen in normal imaging reports. Also, CT scan is not recommended for paediatric and young patients, pregnant females, and patients with a history of contrast allergy. Hence, we recommend doing CRP as the primary laboratory marker for suspected cases of AA. CT abdomen is the ideal imaging modality in cases of suspected AA where clinical examination, laboratory values, and USG are inconclusive. 
